# Physical Activity or Inactivity and Fatigue in Children and Adolescents: A Cross-Sectional Study

**DOI:** 10.3390/jcm14051574

**Published:** 2025-02-26

**Authors:** Véronique-Aurélie Bricout, Duy-Thai Nguyen, Anne Favre Juvin

**Affiliations:** 1 HP2, University Grenoble Alpes, F-38000 Grenoble, France; 2 INSERM U1300, HP2, F-38000 Grenoble, France; 3 CHU de Grenoble, UM Sports et Pathologies, F-38000 Grenoble, France; annefavrejuvin@gmail.com; 4 UM Sports et Pathologies, CHU Sud, CS 90338, Avenue de Kimberley, F-38434 Echirolles, France; 5 Clinical Research Committee, Vietnam Society of Sleep Medicine (VSSM), Dalat 660000, Vietnam; dr.thaind@gmail.com; 6 National Institute for Control of Vaccines and Biologicals, Ministry of Health, Hanoi 100000, Vietnam

**Keywords:** fatigue, adolescent health, questionnaire, physical activity or inactivity

## Abstract

**Background/Objectives**: Physical inactivity needs to be strategically addressed throughout adolescence because it is often accompanied by an alteration in the quality of life. Fatigue is one of the most common symptoms encountered in medical practice. However, little is known about the causal relationship between lifestyle and fatigue. The purpose of this study was to describe the prevalence of fatigue among children and adolescents and to investigate whether various sport types and training volume/week are related to increased fatigue over time while considering gender and age. **Methods**: A total of 1617 participants (mean age = 13.3 ± 1.9 years, 8.2 to 16.1) completed a questionnaire assessing their perception of fatigue and including questions regarding weekly physical practices and attitudes toward sports and health. The participants were secondary school students, whether or not they practiced sports, leisure activities, or music. Analyses of variance and Newman–Keul post-hoc tests were applied to assess differences between groups. A mediation model was used to determine the relationship between physical activity and fatigue score with the relevant role of mediating factors (physical activity level or physical activity type). **Results**: Older adolescents had significantly higher scores of fatigue than younger ones (28.9 ± 17.5 vs. 25.6 ± 16.6; *p* < 0.01). Girls had significantly higher scores of fatigue than boys (30.7 ± 17.5 vs. 23.8 ± 15.8; *p* < 0.001). Additionally, 10.7% of participants were sedentary and presented significantly higher scores of fatigue than participants in the Leisure (35.1 ± 18.2 vs. 29.4 ± 17.3; *p* < 0.001) and Sport groups (35.1 ± 18.2 vs. 25.3 ± 16.2; *p* < 0.0001). In the sedentary group, low physical activity was associated with a high score of fatigue; but in the Sport group, high physical activity was associated with a low score of fatigue. **Conclusions**: Fatigue is closely related to health, physical activity volume, gender, and age. Fatigue symptoms obtained through a questionnaire assessment can serve as an important index for health evaluation.

## 1. Introduction

Physical inactivity has become the most significant public health problem of the 21st century, and there are concerns that young people are more sedentary than previous generations [1]. Numerous well-conducted prospective observational studies have demonstrated that individuals who are the least active and least fit face the greatest risk of developing a variety of chronic diseases, such as heart disease, diabetes, and obesity [2], and an increased risk of all-cause mortality [3].

Fatigue has been recognized as a useful health index in the daily lives of adolescents [4,5]. The evaluation of the fatigue state is essential for understanding the living habits of adolescents and practicing healthy activities. Nevertheless, while fatigue is typically reported in numerous studies, its definition may significantly vary according to the study country or cultural specificities [6]. Fatigue comes in various expressions. Acute fatigue is a normal, protective mechanism in healthy individuals. It is usually linked to a single cause and is often relieved by rest or lifestyle changes. Sustainable fatigue (or chronic) is considered maladaptive or pathologic, lasts 6 months or more, adversely affects physical and mental function, and may have multiple and/or unknown causes [5,6]. This state decreases the ability of individuals to perform a particular task by altering alertness, vigilance, and motivation. Nowadays, there are no biological markers and/or indicators for rapidly diagnosing a state of fatigue with great precision. In the international literature, there are a very large number of questionnaires to assess fatigue, and the almost systematic use of fatigue evaluation questionnaires in adult populations has shown the relevance of these diagnostic tools for truly revealing physiological maladaptations or disorders appearing in this state [7]. In children or adolescents, fatigue is thus a complaint that deserves to be taken into consideration because it may be the only manifestation of an underlying organic disorder; it is, therefore, important to monitor fatigue evolution. The difficulty of evaluating fatigue is not only due to its subjectivity in meaning and experience but also due to its multidimensional components and heterogeneous nature. The key problem is moving from the patient’s subjectivity to a more objective approach; the challenge is to rationalize a symptom at the crossroads of several diseases and specialties. Previous cross-sectional studies have shown that fatigue, including sustainable fatigue, is associated with age, gender, physical activity (PA), and socioeconomic status [8,9,10]. In a sport context, the French Society of Sports Medicine strongly recommends the systematic use of a now-validated questionnaire in children [11], which has also shown its sensitivity according to age, gender, and sport activity [9].

Low levels of exercise are linked to high levels of physical fatigue [12,13]. However, it has not been shown whether there exists a link between the onset of fatigue and the level of PA or inactivity in youth. Although numerous studies have demonstrated the benefits of increased PA in reducing chronic diseases in the general adult population, little is known regarding the association between health-related quality of life and PA level. In this context, participation in regular PA has been proposed to increase physical fitness [3,12], modify the quality of life [3,12], and positively influence emotional well-being [3,12]. Many young people do not meet the current recommendations for PA every day [1], and a majority of them are adopting increasingly sedentary behavior [1].

The aim of the present study is to examine the associations between perceived fatigue, as evaluated through a questionnaire on fatigue, and physical activity. The relationship between subjective fatigue symptoms and different factors characterizing adolescents has been examined (gender, age, physical training volume, disease, or acute pathology).

## 2. Materials and Methods

The design was a prospective observational study (Strobe checklist included in Supplementary File).

### 2.1. Participants

Participants included in this study were secondary school students, whether or not they practiced sports, leisure activities, or music. All participants lived in the Auvergne Rhône Alpes region (France) and came from two metropolitan areas (Grenoble and Albertville), and they were aged 8–17 years. Informed consent was obtained from each subject after a full explanation of the project. The parents of each participant gave their consent to take part in this study. Each subject signed his or her consent in the presence of a member of the research team, with whom he or she could ask any questions. The experimental protocol in this study was approved by the authorities of the schools where the students completed the questionnaire. The medical service of the Rector of the Academy, under the authority of the Ministry of Education, has also validated the use of this questionnaire.

A total of 1650 respondents completed the survey. Only 1617 participants were retained (Table 1). Subjects were primary (n = 297), middle (n = 782), and high-school students (n = 538), recruited from schools serving diverse populations (Auvergne Rhône Alpes, France). Participants completed the questionnaire once, including questions regarding weekly physical practices and attitudes toward sports, sleep, and health. The questionnaire was filled in using a method that was always the same: at school during science lessons with a member of the research team. For athletes, the questionnaire was completed before a training session, in the presence of only one member of the research team, to avoid any influence from the sports coach on the answers. The survey took 3 years to complete.

Several groups were created for our analysis according to the following classifications, depending on age, physical activity level, volume training per week, sports, and health problems.
**Age group:**
group 1 (G1): group 2 (G2): and group 3 (G3): 8 to <12 years old >12 to <14 years old >14 years old**Physical activity level:**INA LPA SportInactive participants: making no PA or less than 1 h per week Leisure Physical Activity: subjects practicing a PA during 1 h 30 to 3 h/week Subjects practicing regularly in a PA within a federal structure during 3 h 30 to more than 15 h/week**Training volume per week:**Train Vol. 1 Train Vol. 2 Train Vol. 3 Train Vol. 4 Train Vol. 53 h 30 → 6 h 6 h 30 → 9 h 9 h 30 → 12 h 12 h 30 → 15 h >15 h**Sports or cultural activity:**Musicians *AthleticsAlpine skiingCross country skiingGymnasticsSwimmingRacket sportFighting sportsDanceTeam sportsOther sportsParticipants in class specialized in music, and having between 4 to 10 h of music practice/week Athletics, Orienteering course; Triathlon. Aerobic; Rhythmic gymnastic; Artistic gymnastic; Trampoline. Competitive swimming; Synchronized swimming. Table tennis; Tennis; Badminton. Judo; Taekwondo; Boxing. Ballet; modern; jazz; hip-hop. Basketball; Football; Hockey; Ice hockey; Handball; Volleyball; Water polo; Rugby. Automobile race; Moto trial; Fencing; Road cycling; Equestrian**Health problems:**InfectionOtolaryngologyTraumatologyGastroenterologyFamily factorsUrinary tract infection, conjunctivitis, skin infection, influenza. Colds, sore throat, sinusitis, rhinitis, cough, and otitis. Sprain, dislocation, fracture, tendinitis. Gastroenteritis, diarrhea, dyspepsia. Relationship problems with parents or siblings, unemployment of a parent, death, and divorce. Scholar difficulties.* The musicians were not involved in competitive or leisure sports. This group was not used to analyze the effects of training volume on fatigue scores.


### 2.2. Questionnaire

The QFES questionnaire is a 2-week recall questionnaire (in French: *Questionnaire de Fatigue de l’Enfant Sportif*). With this questionnaire, we can quickly identify a state of unfitness with a score higher than 45/120, the threshold beyond which fatigue must be monitored [11]. The fatigue score is calculated by summing the scores of 30 items, each evaluated by the child on a 5-point Likert scale (0 to 4 points). These 30 items represent two domains (physical and psychological) across seven dimensions characterizing fatigue: (1) sports performance; (2) symptoms; (3) sleep and appetite; (4) motivation; (5) attention and concentration; (6) relational behavior; (7) anxiety and self-reliance [11]. The procedures for completing the QFES are specified elsewhere [11], as well as those for determining the fatigue score. In an initial work, the questionnaire was evaluated for reliability, sensibility, and specificity [11].

### 2.3. Sampling and Procedure

The QFES fatigue questionnaire is a rapidly implemented, easy-to-use, standardized tool for the evaluation of seven broad dimensions, each of which contains several items. The procedure for completing the QFES is accurate: participants completed the questionnaire in a school setting under the supervision of a teacher, or in a sports association under the supervision of a research assistant, who ensured that the data were correctly collected to avoid missing data.

### 2.4. Statistics

Results are given as means ± SD. The normality of the fatigue score distribution was assessed with a Kolmogorov–Smirnov test. To compare physical activity based on categories of sport participation, participants were divided into three groups (INA, LPA, and Sport groups) and analyzed using analysis of variance (ANOVA). A Newman–Keul post-hoc test was used to examine any differences with respect to age, gender, sport activity, or pathology. To determine the independent effects of age, sex, and physical activity level on fatigue score, we conducted a multivariable regression analysis with fatigue score as the dependent variable (Statistical software: R version 4.4.2). A mediation model (Baron and Kenny procedure and bootstrap mediation) was applied. The mediation model tested whether training volume mediates the effect of physical activity level on fatigue score. Hypothesized mediation model:

- Independent variable (IV): level of physical activity
- Mediator (M): training volume
- Dependent variable (DV): fatigue score

The mediation effect was assessed using Baron and Kenny’s steps:

1. Testing the effect of the IV on the DV.
2. Testing the effect of the IV on the mediator.
3. Testing the effect of the mediator on the DV while controlling for the IV.
4. Testing the indirect effect using bootstrap confidence intervals (5000 iterations).

A significant indirect effect would confirm that training volume plays a mediating role between physical activity level and fatigue score. The statistical significance threshold was set at *p* < 0.05.

## 3. Results

Overall, 1617 out of 1650 respondents completed the survey, which corresponded to a response rate of 98.0%. Individuals who failed to provide complete data for the measures (*n* = 33) did not differ significantly in terms of age, gender, or PA from those who provided data for analysis, but they refused to participate for personal reasons, disinterest, or incomplete answers.

The mean age of participants was 13.3 ± 1.9 years (range 8.2 to 16.1). Among the three age groups, older adolescents (G3) had significantly higher scores of fatigue than younger participants (G1 and G2, *p* < 0.004; Table 1). The sample of this study showed a homogenous distribution between girls and boys (49.2% male vs. 50.8% female; Table 1), but girls had significantly higher scores of fatigue than boys (*p* < 0.0001). Across all groups, being a girl was related to a higher score of fatigue than being a boy (r^2^ = 0.663, *p* < 0.01).

Among participants, 10.7% were inactive, 22.3% engaged in leisure activities, and 67% practiced sport activities. The INA group presented a significantly higher score of fatigue compared to the LPA (*p* < 0.001) and Sport groups (*p* < 0.0001). In this INA group, 62.5% were female, and the older the children are, the more likely they are to become inactive (Table 1), especially among girls in grade 16 (Table 1).

**Figure 1:**
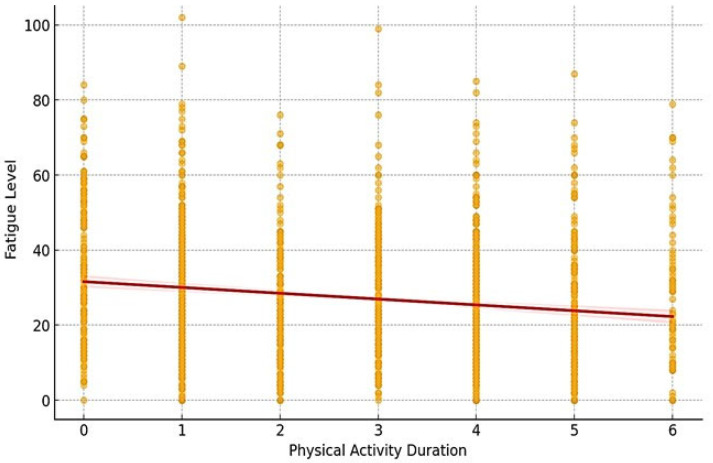
Mediation effect: training volume effect on fatigue score. 0 = INA group; 1 = LPA group; 2 = Train Vol. 1 (3 h 30 → 6 h); 3 = Train Vol. 2 (6 h 30 → 9 h); 4 = Train Vol. 3 (9 h 30 → 12 h); 5 = Train Vol. 4 (12 h 30 → 15 h); 6 = Train Vol. 5 (>15 h).

According to PA level, a score of fatigue increased or decreased depending on the group: in the INA group, low physical activity was associated with a high score of fatigue; but in the Sport group, conversely, high physical activity was associated with a low score of fatigue. This result was confirmed using the regression plot obtained from the mediation model. This plot demonstrated the mediating effect of activity duration on fatigue score (Figure 1). The red regression line highlighted the overall trend: an increase in physical activity duration was associated with a reduction in fatigue scores. Age (*p* < 0.001), female sex (*p* < 0.001), and being in the leisure or Sport groups (both *p* < 0.01) were all significant predictors. Specifically, each additional year of age was associated with a 0.41-point increase in fatigue score (95% CI: 0.20, 0.62). Compared to males, females had a mean score of 2.93 points higher (95% CI: 1.93, 3.93). Meanwhile, inactive participants had the highest fatigue scores; those in the Sport group scored, on average, 3.51 points lower (95% CI: −4.50, −2.51) than the inactive group, explaining approximately 22.3% of the variance in fatigue scores 0.217). Residual diagnostics indicated no major violations of regression assumptions. This supports the mediation analysis findings that activity duration plays a critical role in mitigating fatigue. Depending on the sport type, the score of fatigue was significantly higher in athletes compared to Skiing/Team sports/Fighting sports/Racket sports/Dance and other sports (*p* < 0.001, Table 1); and significantly lower in racket sports compared to Swimming/Gymnastics/and Musicians (*p* < 0.001, Table 1).

In the total population, the score of fatigue increased significantly when participants reported a health problem (*p* < 0.01). The largest score of fatigue increase was left with family problems compared to the ‘no problem group’ (*p* < 0.0001; Table 2). The ‘Otolaryngology’ component was the most frequently selected across the entire population, as in INA, LPA, and Sport groups. Specifically, in each group, another component was frequently selected by the subjects: the ‘Traumatology’ component in INA (8%), and in the Sport group, ‘Infection’ (4.8%) and ‘Traumatology’ (4.7%).

**Figure 2:**
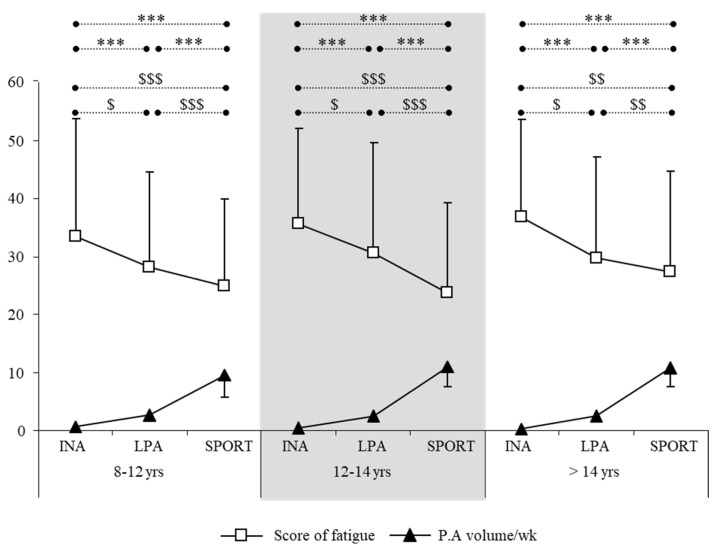
Score of fatigue and PA volume by age group. PA volume significantly different: *** *p* < 0.001. Score of fatigue significantly different: $ *p* < 0.05; $$ *p* < 0.01; $$$ *p* < 0.001.

According to PA level and age group, the score of fatigue was lower in the Sport group compared to LPA and SED. In the INA group, low physical activity was still associated with a high score of fatigue (Figure 2).

## 4. Discussion

The aim of the present study is to examine associations between perceived fatigue and physical activity as evaluated using a questionnaire. The relationship between subjective fatigue symptoms and various factors characterizing adolescents has been examined (gender, age, physical activity training volume, disease, or acute pathology).

The primary objective of this work was to show that in the field of health behavior, the use of questionnaires assessing perceived fatigue is of interest for evaluating adolescent health behaviors and the determinants of fatigue in order to promote physical activity. As advocated by Bricout et al. [9], such public health approaches also represent a meaningful complement to strategies targeting high-risk sedentary populations.

In our study, 10.7% (n = 173 INA) of young people do not engage in sufficient PA, with a volume between 0 and 1 h/week, which is very inadequate and far below the minimum recommendation of 30 min moderate-intensity PA daily (WHO recommendations) [14]. Physical inactivity has become a major public health problem, particularly among young people [1], and in the INA group, by the age of 12 years, an increasing number of children become more inactive and cease all physical activity. The general decrease in activity is concerning, given the role PA plays in reducing the risk of many chronic health conditions. Several theories have been proposed to help explain changes in PA levels among youth over time [15], and it seems to be environmental and social factors that explain this decrease. Previous studies have shown that reported PA declines with age in both genders [16,17]. In our work, we observed this same decline with age, specifically among girls (Figure 1). Nevertheless, girls have been shown to be less active than boys, and we also observed this result in our study, with twice more inactive girls than boys (62.5% vs. 37.5%, respectively). Additionally, a larger proportion of girls were thus totally inactive (0 h/wk) compared to boys. The Youth Risk Behavior Surveillance System survey of high school students in the United States estimated that the percentage of girls who participate in sufficient vigorous physical activity declines from 63.6% in ninth grade to 46.4% in 12th grade [17,18]. The reasons that girls are less physically active than boys are not clear, but in the literature, gender was associated with physical activity in 81% of studies on children, and in studies on adolescents or pediatric studies [18]. Explanations for these gender differences in adolescents’ PA are likely multifactorial: girls may have lower motivation to practice exercise, lower perceptions of the health benefits of practice, and less enjoyment of sports compared to boys [19]. Some environmental and cultural factors have also been proposed as barriers that discourage PA among girls [19,20]. The literature also reports gender differences in physiological responses to physical activity. In particular, exercise tolerance thresholds and global exercise capacity [1] are lower in girls. Consequently, the type of PA, in terms of frequency, duration, intensity, and type of exercise may vary between girls and boys, with different health benefits. The mechanisms underlying these gender differences in response to PA in brain health [21] and neurocognitive health [21] have been reported in the literature. The latest work highlights the role of neurotrophic factors, in particular brain-derived neurotrophic factor (BDNF), in improving cognition, brain function, and neuroplasticity induced by physical activity. However, the role of BDNF has not been explored in children or adolescents, but its impact on older adults has been extensively studied. Nor has the link between these neurotrophic factors and fatigue been studied to date. It is therefore possible that different pathophysiological mechanisms may be at the root of fatigue in children and adolescents. An approach that takes these gender differences into account is needed to better understand the expression of fatigue and develop personalized management strategies.

Paradoxically, adolescents who showed the lowest PA volume per week are those who had the highest score of fatigue and although girls were less active than boys, they showed higher fatigue scores (Figure 1). This tendency of girls to present higher fatigue scores has been found in previous work [9]. The dose-response relationship between fatigue-based inactive behavior and health suggests that the more time adolescent girls spend engaging in sedentary activities, the greater the physical, behavioral, and psychosocial health consequences. Consistent with emerging literature [19,22], screen time in adolescent girls is associated with weight status, energy intake, depression, and musculoskeletal pain, independent of physical activity levels. Furthermore, a consistent inverse association between screen time and physical activity has been reported. In our study, we also observed that the “health problem” variable was lowest among adolescents engaged in sports. In the INA group, the item “fatigue” has the highest score. These results confirm the beneficial effect of the practice on the feeling of fatigue. Some hypotheses propose the physiological effect of physical activity on monoamines in the brain affecting neurotransmission or on the production of endorphins and their pain reduction and euphoric effects [23]. Other positive effects of PA have been reported and may explain the increase in fatigue and health problems in the INA group. Thus, with physical inactivity, muscle mass decreases, with a loss of muscle strength but an increase in fat mass [13]. Muscle capillary density declines along with mitochondrial enzymatic activity and Adenosine triphosphate production (ATP). This leads to a loss of muscle oxidative potential and increased fatigability of muscle. This, in combination with loss of cardiac output, leads to a decrease in VO_2max_ (maximal aerobic capacity) [13]. In response to unloading, bone strength decreases through a rapid and sustained increase in bone resorption and a more subtle decrease in bone formation [13]. Furthermore, PA has been associated with better mental health, a positive self-concept, and a reduction in anxiety and depression in adolescents [5,23]. A lack of physical activity has been linked to poor mental health and well-being. This result was also observed in our results: all health items showed higher scores in INA compared to the LPA or Sport groups. The most active people would be those who had the lowest risk of developing depression and anxiety, which would be improved by regular AP [5,23].

Another interesting result was observed in our work: older adolescents (G3) had significantly higher scores of fatigue than younger adolescents (G1 and G2, *p* < 0.004). With age, score of fatigue increased (Table 1), and PA volume decreased (Figure 2).

If this association between age and the decline of PA is very well described in the literature [18,19,20], few studies have examined the effect of age on the feeling of fatigue as a function of PA volume. To our knowledge, this is the first study to examine this triple relationship. Some hypotheses have been proposed to explain this result. It might be possible that the effects of schoolwork on the score of fatigue develop very quickly in middle and high school adolescents, for which some workloads are important, and stress induced by the Baccalaureate was an additional constraint. But in all groups, all adolescents were subject to the same schoolwork and achievement requirements, even though some teenagers combine this schoolwork with significant sport training volumes, though with lesser scores of fatigue. We believe that the positive effect of regular PA on fatigue indicators and, therefore, better health of adolescents is expressed here. A recommendation from the American College of Sports Medicine and the American Heart Association regarding PA and public health in adults advises that, in order to promote and maintain health, moderate-intensity aerobic PA for a minimum of 30 min on five days each week, or vigorous-intensity aerobic PA for a minimum of 20 min on three days each week should be carried out.

This observation is further confirmed by the results achieved by sport adolescents who regularly practice in clubs. Indeed, in the ‘Sports’ groups, the scores of fatigue are significantly lower than those of the LPA and INA groups, confirming the beneficial effect of regular PA. However, there would be an ideal training volume, one located around 1 h 30/day. Beyond 15 h/week, fatigue scores begin to rise again. Excess training and physical inactivity are two unsuitable behaviors that are associated with fatigue expression among young people.

Note that this is in sports for which training volumes are known to be very important (Swimming, Gymnastics, and Athletics), fatigue scores are often highest due to the rigorous demands imposed on these young athletes.

We can also specify that in this sample, there were young musicians who were followed and that in this group specifically the young people were subjected to very important constraints: intense practice and very strict selection criteria to gain access to the conservatory. These conditions can be related identically to those of the sport competitors in our sample (those in the Sport 5 group). These athletes were all young athletes coached by a sport federation, a club, or a sport study school, with a view to reaching competition at the highest level. In this group, this meant that the training volume could rise significantly (up to 15 h per week) during certain periods of the sport season, and for them, the evocation of a state of fatigue is translated as a ‘negative performance’, thus the fatigue represents another perception. The same age-related differences were also observed in this group (Figure 2) and are probably linked to “environmental” factors. In fact, 14-year-old sport adolescents have the highest fatigue scores. This may be explained by the stronger demands made by the school at this age (College Diploma examination) and by the constraints of the sport selection process, which enables some children to access high-level French teams. Unfortunately, there are still too few studies on the risks associated with the deleterious effects of intensive sports in general and none on overtraining in child athletes in particular.

This study has some limitations and strengths. One important limitation is that specific information regarding participation in race or socioeconomic status was not collected. This information could have provided more insight into the associations between the types or settings of sport participation and physical activity. Longitudinal studies and (natural) experiments are needed to gain a better understanding of prediction and causal pathways. Another limitation is the reliance on self-reported measures of PA, which may have introduced bias. The analyses were conducted on an urban sample of adolescents. Therefore, care should be taken when translating these results to other populations.

Some strengths of our study should be highlighted. In contrast to previous studies, we studied PA and fatigue; both are closely involved dimensions in the quality of life and health. We used a large and representative sample, covering a broad age range from early to late adolescence. The applied questionnaires were extensively pretested and validated [8,11]. We are not aware of other studies that have tested the possible mediating role of PA on fatigue expression.

Finally, it would also be interesting to explore the results obtained with regard to the latest neuroscience work, which shows the role of certain brain structures (i.e., the cerebellum, hippocampus, and mirror neurons) that are involved in a wide range of functions (motor and observation) and could have an effect on the expression of fatigue.

## 5. Conclusions

To conclude, adolescence is a very important age stage in which the physical fitness of young adults develops markedly, and their lifestyle is determined. Fatigue is closely related to health, physical fitness, and stress, and subjective fatigue symptoms can also be used as an important index for health evaluation. Considering the significance of today’s fatigue evaluations for adolescents, it is indispensable to have simple and rational scales for assessing subjective fatigue symptoms that evaluate fatigue appropriately. In children and teenagers, the most detailed examinations must be prescribed only in very particular clinical situations. Although there are various subjective fatigue scales, it is desirable to have detailed evaluations focusing on the evaluation of fatigue symptoms for adolescents to address them as quickly as possible. Lastly, even though the use of the QFES appears to play a valuable role in the follow-up of young in general, it is especially interesting to apply it during puberty, which is known to be a sensitive period, or in specific populations such as adolescent athletes.

There are public health issues to provide appropriate opportunities to encourage physical activity among children and adolescents. In particular, promoting physical activity to limit the PA decline with age and encouraging PA among young girls is essential. The action plan published by the WHO (The Global Action Plan on Physical Activity: Global action plan on physical activity: more active people for a Healthier World, 2018–2030) provides a set of policy guidelines that can be applied and adapted to a large number of countries. The action plan sets out four strategic objectives and is associated with 20 evidence-based policy actions that can help educate children and adolescents about the health issues associated with regular physical activity.

## Figures and Tables

**Table 1:** Demographic characteristics depending on the age, physical activity level, and training volume per week.

	n	Score of Fatigue	*p* Value
Total	1617	27.3 ± 17.0	
Male	796	23.8 ± 15.8	
Female	821	30.7 ± 17.5	*
G1: 8–12 yrs	464	26.9 ± 16.5	
G2: 12–14 yrs	504	25.6 ± 16.6	
G3: >14 yrs	649	28.9 ± 17.5	$
Inactive (INA)	173	35.1 ± 18.2	
Leisure y (LPA)	361	29.4 ± 17.3	¤
Sport (Sport)	1083	25.3 ± 16.2	¤¤, §
Train Vol. 1	174	24.6 ± 15.7	¤¤, §
Train Vol. 2	233	28.3 ± 16.2	¤¤
Train Vol. 3	392	24.6 ± 15.5	¤¤
Train Vol. 4	213	23.2 ± 16.7	¤¤, §§, Ψ
Train Vol. 5	71	28.3 ± 18.8	¤¤, ¶, λ
Athletics	11	38.9 ± 19.4	
Alpine Skiing	470	24.3 ± 14.8	#
Cross-country skiing	181	26.9 ± 20.4	#
Swimming	17	33.3 ±16.7	£
Gymnastics	20	33.8 ± 14.6	£
Team sports	160	25.5 ± 16.9	#
Fighting sports	73	26.5 ± 15.2	#
Racket sports	53	19.0 ± 12.0	#
Dance	89	26.3 ± 15.5	#
Other sports	36	24.3 ± 14.6	#
Musicians	79	31.3 ± 19.2	£

Significant difference: from male: * *p* < 0.001; from G2: $ *p* < 0.01; from INA: ¤ *p* < 0.001; ¤¤ *p* < 0.0001; from LPA: § *p* < 0.05, §§ *p* < 0.001; from Train Vol. 1: ¶ *p* < 0.05; from Train Vol. 2: Ψ *p* < 0.05; from Train Vol. 4: λ *p* < 0.05; from athletics: # *p* < 0.01; from racket: £ *p* < 0.01.

**Table 2:** Impact of health problems on score of fatigue.

	Entire Sample n = 1617	INA n = 173	LPA n = 361	Sport n = 1083
No problem n (%)	23.8 ± 14.7 1270 (78.5%)	29.9 ± 16.1 120 (69.4%)	25.4 ± 14.6 287 (79.5%)	22.4 ± 14.3 863 (79.7%)
All health problems n (%)	40.1 ± 18.7 *** 347 (21.5%)	47.0 ± 17.4 ££ ** 53 (30.6%)	44.8 ± 18.8 ££ ** 74 (20.5%)	36.8 ± 18.3 * 220 (20.3%)
Traumatology n (%)	43.6 ± 18.8 ** 69 (4.3%)	38.3 ± 15.6 14 (8%)	39.5 ± 5.7 4 (1.1%)	45.4 ± 20.1 51 (4.7%)
Fatigue n (%)	44.9 ± 16.8 ** 43 (2.6%)	51.6 ± 14.2 10 (5.8%)	47.0 ± 16.5 13 (3.6%)	40.3 ± 17.5 20 (1.8%)
Asthma n (%)	45.5 ± 10.6 ** 12 (0.7%)	45.0 ± 14.2 3 (1.7%)	42.8 ± 10.1 6 (1.7%)	51.3 ± 9.2 3 (0.3%)
Infections n (%)	38.8 ± 18.4 ** 71 (4.4%)	52.0 ± 16.6 9 (5.2%)	44.4 ± 19.7 10 (2.8%)	35.5 ± 17.5 52 (4.8%)
Otolaryngology n (%)	34.8 ± 17.1 * 78 (4.8%)	46.8 ± 21.4 10 (5.8%)	42.5 ± 17.4 19 (5.3%)	29.4 ± 13.7 49 (4.5%)
Allergy n (%)	33.1 ± 17.7 * 29 (1.8%)	50.0 ± 20.6 6 (3.5%)	20.2 ± 13.6 5 (1.4%)	31.0 ± 14.0 18 (1.7%)
Gastroenterology n (%)	35.8 ± 18.3 * 21 (1.3%)	0 ± 0 0	46.2 ± 20.5 4 (1.1%)	33.4 ± 17.5 17 (1.6%)
Family problems n (%)	51.4 ± 23.5 *** 24 (1.5%)	65.0 ± 0 ** 1 (0.6%)	57.9 ± 21.1 ** 13 (3.6%)	41.7 ± 25.2 10 (0.9%)

Significant difference from ‘No problems’: * *p* < 0.01; ** *p* < 0.001; ***p < 0.0001. Significant difference from Sport group: ££ *p* < 0.001.

## Data Availability

Data are available on reasonable request from the corresponding author.

## Supplementary Materials

The following supporting information can be downloaded at: https://www.mdpi.com/article/10.3390/jcm14051574/s1, Supplementary file: checklist of items that should be included in reports of observational studies.
